# Amelioration of CCl_4_-Induced Hepatotoxicity in Rabbits by* Lepidium sativum* Seeds

**DOI:** 10.1155/2019/5947234

**Published:** 2019-03-07

**Authors:** Mazin A. Zamzami, Othman A. S. Baothman, Fatma Samy, Mohamed Kamel Abo-Golayel

**Affiliations:** ^1^Biochemistry Department, Faculty of Science, King Abdulaziz University, Jeddah, Saudi Arabia; ^2^Microbial Toxicology & Natural Products Centre, Faculty of Science, King Abdulaziz University, Saudi Arabia; ^3^Pathology Department, Faculty of Medicine, Ain Shams University, Cairo, Egypt; ^4^Medical Research Centre, Ain Shams University Hospitals, Ain Shams University, Cairo, Egypt

## Abstract

The current study aimed to evaluate the probable protective effect of* Lepidium sativum* seeds (LSS) against CCl_4_ induced hepatic injury in New-Zealand rabbits. Rabbits were randomly divided into two main groups; group-A (noninjured group, n=15) was divided to subgroups A1 (untreated control) and A2 and A3 which received 200 & 400 mg/kg bw of LSS, respectively, in their diet daily. Group-B (injured group, n=30) were subcutaneously injected with CCl_4_ (0.5 ml/kg bw) starting from day one of the experiment and were equally divided into 3 subgroups: B1 received normal standard diet and B2 & B3 received 200 & 400 mg/kg bw of LSS, respectively, in their diet daily. Five rabbits of all subgroups were decapitated 5 and 10 weeks after experimental running. Biochemical analysis revealed significant decrease in serum levels of transaminases, *γ*-GT, ALP, total bilirubin, cholesterol, triglycerides associated with significant increase in the serum levels of T protein and albumin of 200 and 400 mg/kg bw of LSS protected rabbits for 5 and 10 weeks as compared with CCl_4_ treated rabbits. Oxidative stress and depressed antioxidant system of the liver tissues were markedly obvious in the CCl_4_ treated group. LSS administration reversed these results towards normalization. Histopathological examination of LSS protected rabbits (200 mg/kg bw of LSS for 10 weeks) showed improvement of the histoarchitectural changes of the liver induced by CCl_4_ to the normal aspect, showing regenerating hepatocytes with no steatosis, discrete chronic venous congestion, and discrete inflammatory infiltrate. The current findings provide new evidence that LSS could reverse the hepatotoxic effects of CCl_4_ and repair the liver functions.

## 1. Introduction

Carbon tetrachloride (CCl_4_) is a xenobiotic industrial solvent that is used to induce chemical hepatitis and liver injuries in experimental animals. Carbon tetrachloride-induced liver injuries are the most common experimental model for monitoring the hepatoprotective activity of certain drugs. A single exposure to CCl_4_ as being a strong hepatotoxic xenobiotic directly leads to severe liver necrosis and steatosis [1, 2]. Mechanistic studies offer evidence that metabolism of CCl_4_ via CYP2E1 to strongly reactive free radical metabolites plays a crucial role in the proposed mode of action. The major metabolites, trichloromethyl (CCl_3_·) and trichloromethyl peroxy (CCl_3_O_2_·) free radicals, are extremely reactive and are capable of covalent bind to cellular macromolecules, preferring fatty acids of the membrane phospholipids. The free radicals induce cell membrane lipid peroxidation via disrupting polyunsaturated fatty acids within these membranes, initiating a sequential free radical chain reaction [1].

*Lepidium sativum *is a wild-growing edible annual herb belongs to Brassicaceae family. It is believed to be originated mainly in Eritrea and Ethiopia. Leaves, roots, and seeds of* Lepidium sativum *are great profitable crops; however, the crop is basically cultivated for seeds. In India, this herb is known as Asalio and it is used in folk medicine since ancient eras [3, 4].* Lepidium sativum *seeds (LSS) exert hypoglycaemic activity both in diabetic and normal rats without interfering with insulin secretion [5]. The seeds are recognized to improve the symptoms of asthma and recover lung function in asthmatic patients [6]. In folk medicine, several parts of* Lepidium sativum* have been utilized for the treatment of liver problems, jaundice, spleen diseases, menstrual problems, gastrointestinal disorders, bone fracture, and arthritis [7–9]. Phytochemical research studies of* Lepidium sativum* demonstrated the existence of flavonoids, glucosinolates, tannins, alkaloids, triterpenes, sterols, benzyl isothiocyanate [10, 11] that were stated to possess analgesic, anti-inflammatory activities, antioxidant capacities, and hepatoprotective characters [10, 12–14]. Although previous reports revealed that hepatoprotective efficacy of LSS against doxorubicin [15] and CCl_4_ [12] induced intoxication has been described, up to our knowledge no studies have been conducted on the LSS existing in the Arabian region. Moreover, the uniqueness of this study is that the active ingredients of LSS extract could be responsible for the bioactivity of these seeds. Therefore, the current study was designed to investigate the probable protective effect of* Lepidium sativum* seeds against CCl_4_ induced hepatotoxicity in New-Zealand rabbits.

## 2. Materials and Methods

### 2.1. Preparation of* Lepidium sativum* Seeds (LSS) Aqueous Extract

*Lepidium sativum* seeds were gained from Alquaseem market of traditional medicine, KSA, recognized and identified by a professor of taxonomy, and deposited at the Herbarium of Biological Department, Faculty of Science, King Abdulaziz University. Before extraction, LSS were washed using double deionized distilled water, dried, and crushed by pestle and mortar. The seeds were permitted to dry in the sunlight for 2 days then homogenized to a fine powder and kept in free-moisture impervious container until use.

### 2.2. Chromatographic Analysis of LSS Using GC-MS

Chromatographic analysis using GC-MS was carried out (Agilent Technologies 7890B GC Systems combined with 5977A Mass Selective Detector). Capillary column (HP-5MS Capillary; 30.0m×0.25mm ID×0.25*μ*m film) and helium as carrier gas were used at a rate of flow of 1.9 ml/min with 1*μ*l injection. The sample was analyzed with the column held initially for 3 min at 40°C after injection, then the temperature was increased to 300°C with a 20°C/min heating ramp, with a 4.0 min hold. Injection was carried out in split-less mode at 300°C. MS scan range was (*m*/*z*): 50–550 atomic mass units (AMU) under electron impact (EI) ionization (70 eV).

### 2.3. Preparation of Fatty Acid Methyl Esters

Fatty acid methyl esters were prepared from different sources using commercial aqueous HCl [16]. Yields of FAME were similar to those obtained with boron trifluoride method. Additionally, the reagent was very safe and appropriate. Shortly, the reagent was made from 9.7 ml commercial concentrated HCI (35% w/w) diluted with 41.5 ml of methanol and was stored in a refrigerator.

A lipid sample was dissolved in 0.20 ml of toluene; then, 1.50 ml of methanol and 0.30 ml of the reagent solution were added in this order. The tube was vortexed and then heated at 100°C for 1 h. After cooling, 1 ml of hexane and 1 ml of water were added for extraction of methyl esters in the hexane phase.

### 2.4. Silylation Agent: BSA. N, O-Bis (Trimethylsilyl) Acetamide

The reaction was carried out by adding 100 uL of BSA + amount of the sample after extraction and heating in water bath at 70°C for two hours and after that injected into GC/MS under the above conditions.

The constituents were determined by mass fragmentations with the NIST mass spectral search program for the NIST/EPA/NIH mass spectral library Version 2.2 (Jun 2014).

### 2.5. Animal Model

Forty-five adult male New-Zealand White rabbits (*Oryctolagus cuniculus*) of 6 months of age and weighing 3-4 kg were included in this study. Rabbits were purchased from the animal house of King Fahd Medical Research Centre (KFMRC), King Abdulaziz University, KSA. Rabbits were maintained for Care and Use of Laboratory Animals according to the criteria of US National Institutes of Health (NIH Publication No 8523, revised 1985) [17].

### 2.6. Treatment of Rabbits, Induction of Hepatic Injury, and Experimental Design

Rabbits were housed in well-ventilated polypropylene cages with husk beds. Rabbits were left to acclimatize to the laboratory conditions (26-28C°, 60-80% relative humidity, 12 h light/dark cycle) for 10 days prior to starting the experimental running during which they received standard diet and tap water ad libitum [18, 19]. Rabbits were divided into 2 groups: normal control group (group -A) and hepatic injured group (group-B) which was subdivided into the following.

Group-A (n:15): five rabbits (Subgroup-A1) out of group-A received normal diet without exposure to CCl_4_ hepatic intoxication to serve as negative control group. Five rabbits (Subgroup-A2) received 200 mg/kg bw of* Lepidium sativum L* seeds in their diet on daily basis till the end of the experiment. Five rabbits (Subgroup-A3) received 400 mg/kg bw of* Lepidium sativum* L seeds in their diet on daily basis till the end of the experiment [20]. Group-B (n:30): all rabbits of this group were subcutaneously injected with CCl_4_ starting from day one of the experiment at a dose of 0.5 ml/Kg bw (20% CCl_4_ in paraffin oil) of previously prepared CCl_4_ [21, 22]. Rabbits of group-B received normal diet and were weighted twice per week to precisely determine the CCl_4_ dose. Also, they were subdivided as follows:

  Subgroup-B1: ten rabbits served as a control pollutant group (+ve control).
  Subgroup-B2: ten rabbits received 200 mg/kg bw of LSS mixed with their food daily.
  Subgroup-B3: ten rabbits received 400 mg/kg bw of LSS mixed with their food daily.
  Five rabbits out of each subgroup of group-B were decapitated 5 & 10 weeks after experimental running.

Diet, hygienic conditions, and behaviour were observed and followed up on daily basis. Rabbits and food were observed ensuring that rabbits ate all of their food before addition of extra seeds in their new meal according to the experimental design. At the end of the 5^th^ and 10^th^ weeks of the experiment, rabbits were fasted 12-16 hours before sacrificing. At the end of 5^th^ and 10^th^ weeks of the study, blood sample from each target rabbits according to the experimental design was withdrawn from the ear vein, collected in a centrifuge tube and kept at room temperature for 20 minutes. Sera were separated by a cooling centrifuge at 3000 rpm for 15 minutes and stored at -80°C until recall. Then, target rabbits were decapitated and the abdomen of each rabbit was excised immediately after scarifying. The liver of each rabbit was removed and divided into 3 segments; 1^st^ segment was immersed directly into 10% formaldehyde solution and then processed for histopathological preparation and examination, the 2^nd^ segment was used for DNA extraction to study the liver DNA integrity, and the 3^rd^ segment was used for preparation of liver homogenate.

Sera of the studied rabbits were used to estimate the levels of ALT, AST, *γ*-GT, ALP, T. Bilirubin, T. Protein, Albumin, Cholesterol, and Triglycerides which have been purchased from (EGY-CHEM for lab technology, Bader City, Egypt). The homogenization of pancreas tissues was carried out according to Gackowski et al. [23]. Liver tissue homogenate of each sample was used to estimate the levels of the antioxidant enzymes: Catalase (CAT), glutathione peroxidase (GPX), Glutathione reductase (GR), Glutathione-S-transferase (GST), and Super-oxide dismutase (SOD) as well as (MDA) for all the studied rabbits using kits from (Bioassay Technology Laboratory, SHANGHAI KORIAN BIOTECH Co.).

### 2.7. Extraction of DNA of Rabbits^,^ Hepatic Tissues and Gel Electrophoresis

We studied the genomic DNA integrity of the liver of all the studied rabbits. In accordance with the purification protocol of total DNA from rabbits^,^ tissues, DNA extraction was processed using (Spin-Column Protocol, QIAGEN Group). Agarose gel (2% agarose gel in 1x TAE buffer) was prepared according to Raj Kumar [24].

### 2.8. Histopathological Examination

Liver tissues were dissected from scarified rabbits, removed, and fixed in 10% formalin solution. The fixed specimens were then trimmed, washed, and dehydrated in ascending grades of alcohol. These specimens were cleared in xylene, embedded in paraffin, sectioned at 4-5 *μ* of thickness, and stained with Hematoxylin and Eosin, then examined microscopically [25].

### 2.9. Statistical Analysis

Analyzing the data was conducted by statistical package software (SPSS). The t-test of significance was tested for identifying the differences between the means. Results were expressed as mean ± SEM.

## 3. Results

### 3.1. GC-MS Analysis of LSS Extract

**Figure 1:**
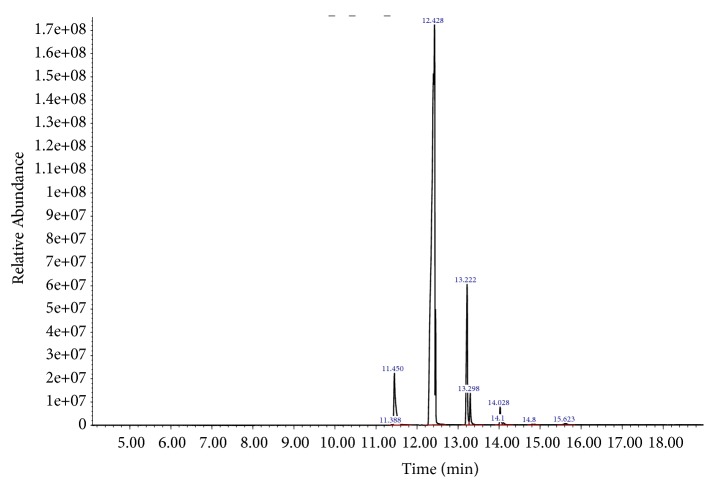
Gas Chromatography Mass Spectrometry (GC-MS) analysis of fatty acids in LSS.

**Figure 2:**
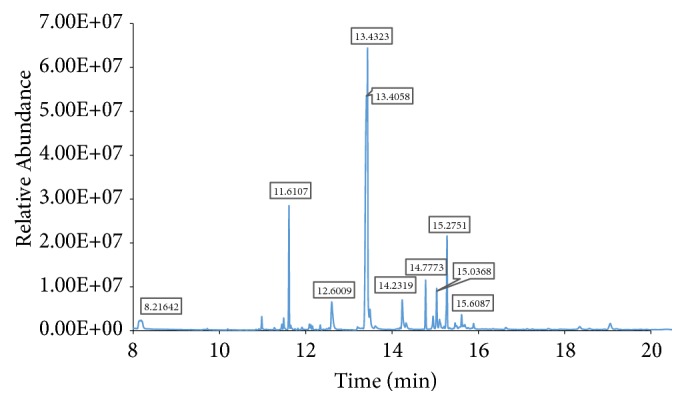
Gas Chromatography Mass Spectrometry (GC-MS) analysis of polyphenols and carbohydrates of LSS.

By comparing the mass spectra of the components with the NIST library, ten major fatty acids peaks were obtained out of which thirty-eight phytocomponents in LSS were described and recognized as shown in Figure 1. These ten major fatty acids were E-10-Methyl-11-tetradecen-1-ol acetate, Hexadecanoic acid, methyl ester, 9,12,15-Octadecatrienoic acid, methyl ester, cis-Methyl 11-eicosenoate, Methyl 18-methylnonadecanoate, Methyl 11-docosenoate, Docosanoic acid, methyl ester, Octadecanoic acid, 9,10-dihydrox, methyl ester, bis(trifluoroacetate), Tetracosanoic acid, methyl ester, Benzoic acid, 3,5-dicyclohexyl-4-hydroxy-, methyl ester. The retention time, peak area, chemical/formulae and molecular weight and M/Z ratio of all fatty acids found in LSS are depicted in Table 1. Similarly, the chemical pattern of LSS extract of polyphenols and carbohydrates revealed by GC-MS analyses exhibited 15 compounds were shown in Figure 2. These active compounds were Glycerol, 3TMS derivative, D-Pinitol, pentakis(trimethylsilyl) ether, Palmitic Acid, TMS derivative, 11-Octadecenoic acid, (Z)-, TMS derivative, alpha.-Linolenic acid, TMS derivative, Stearic acid, TMS derivative, Sinapinic acid, 2TMS derivative, 11-Eicosenoic acid, (Z)-, TMS derivative, Sucrose, 8TMS derivative, 3,7,11,15-tetramethylhexadecan-1,3-diol, silylated, Sucrose, 8TMS derivative, Behenic acid, TMS derivative, D-(+)-Turanose, octakis(trimethylsilyl) ether and beta.-Sitosterol, TMS derivative. The retention time, peak area, chemical/formulae and molecular weight and M/Z ratio of polyphenols and carbohydrates found in LSS are depicted in Table 2.

### 3.2. Assessment of Biochemical Markers

**Figure 3:**
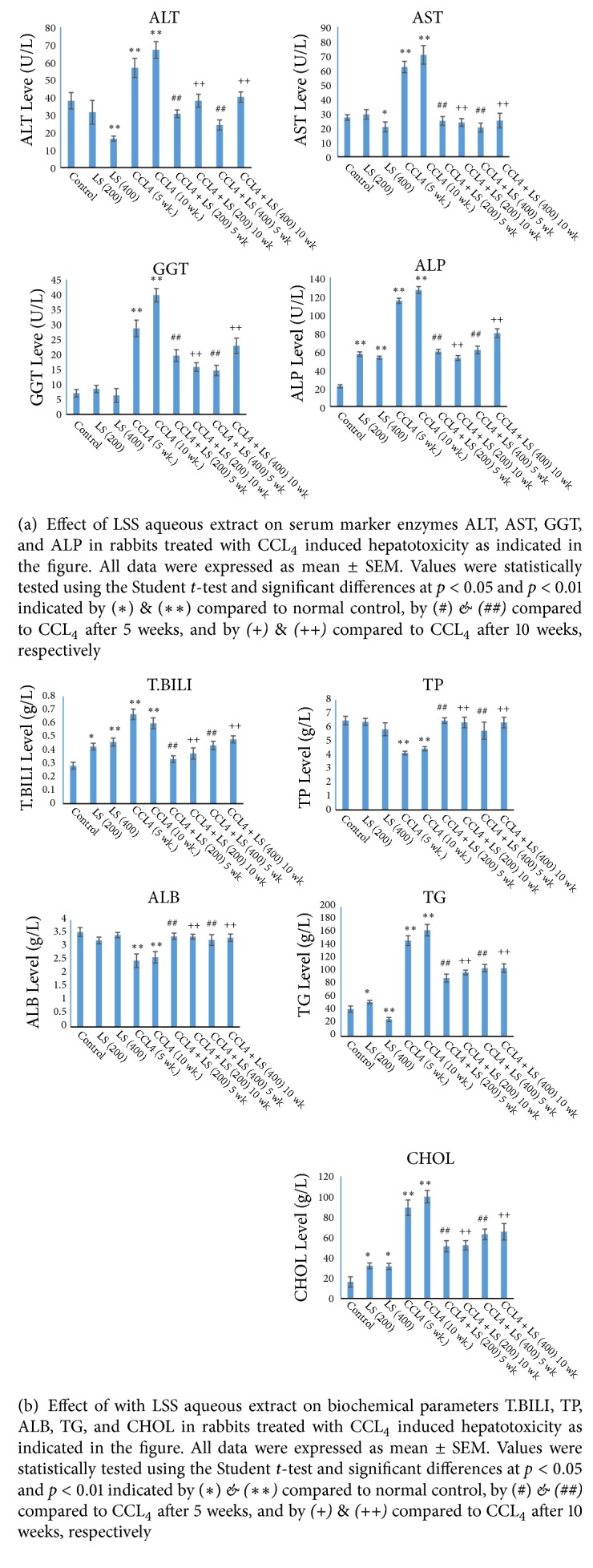


As shown in Table 3 & Figure 3(a) serum levels of liver biomarkers ALT, AST, *γ*-GT, and ALP activities were significantly elevated (P≤ 0.01) with percentages of 49%, 128%, 310%, and 421%, respectively, in rabbits injected with CCl_4_ for 5 weeks and for 10 weeks with percentages of 76%, 159%, 468%, and 474%, respectively. Serum total bilirubin, triglycerides, and cholesterol levels were also significantly elevated (P≤ 0.01) in rabbits injected with CCl_4_ for 5 weeks with percentages of 139%, 250%, and 72%, respectively, and for 10 weeks with percentages of 114%, 290%, and 512%, respectively, compared to those of the normal control (Figure 3(b)). In addition, serum total protein and albumin levels were significantly decreased (P≤ 0.01) in rabbits injected with CCl_4_ for 5 weeks with percentages of 36% and 30%, respectively, and for 10 weeks as well with percentages of 31% and 26%, respectively, compared to those of the normal control. Serum ALT, AST,***γ***-GT, ALP, total bilirubin, triglycerides, and cholesterol activities were significantly reduced (P≤ 0.01) in rabbits protected with 200 mg /kg bw of LSS with percentages of 46%, 60%, 31%, 48%, 50%, 39%, and 42%, respectively, and for 400 mg/kg bw with percentages of 57%, 67%, 49%, 46%, 34%, 29%, and 28%, respectively, for 5 weeks compared to the corresponding CCl_4_ treated group (Table 3 and Figures 3(a) and 3(b)). Serum ALT, AST, **γ**-GT, ALP, total bilirubin, triglycerides, and cholesterol levels were also significantly reduced in rabbits protected with 200 and 400 mg/kg bw of LSS for 10 weeks compared to those of the corresponding CCl_4_ treated group as shown in Table 3 and Figures 3(a) and 3(b).

As shown in Table 3 and Figure 3(b) serum total protein and albumin levels were significantly elevated (P≤ 0.01) with percentages of 57%, 37% in 200 mg/kg bw of LSS protected group and 39%, 31% in 400 mg/kg bw of LSS protected group for five consecutive weeks compared to those of the corresponding CCl_4_ treated group, respectively. Same behaviour was noticed after 10 consecutive weeks of CCL_4_ treatment accompanied with LSS protection; the elevation was 43%, 29% in 200 mg/kg bw of LSS protected group and 42%, 27% and in 400 mg/kg bw of LSS protected group compared to those of the corresponding CCl_4_ treated group, respectively.

### 3.3. Assessment of Oxidative Stress and Antioxidant Activity

**Figure 4:**
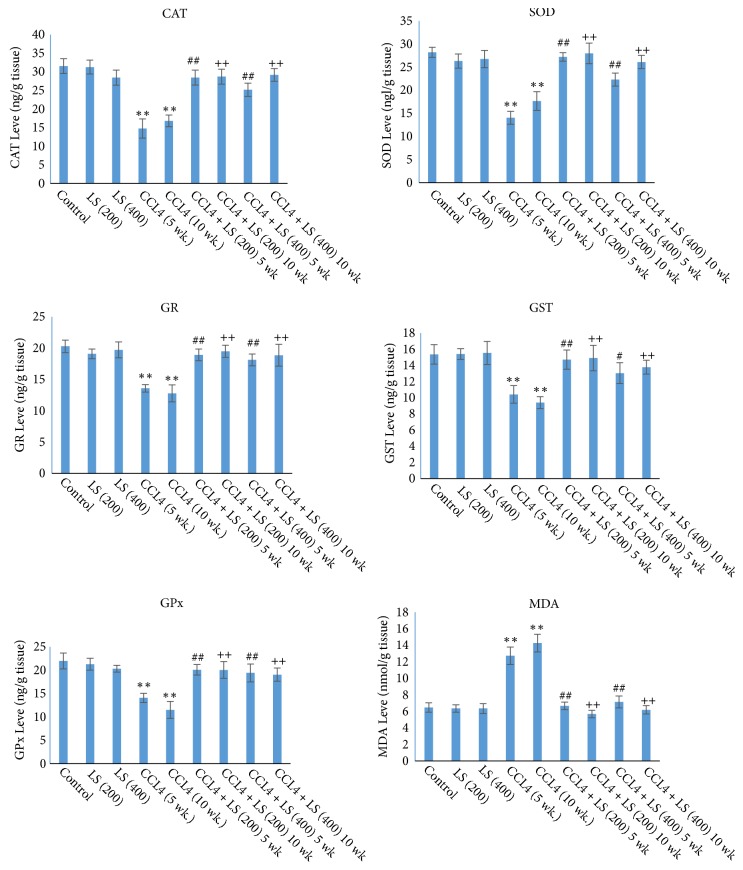
Effects of LSS on oxidative stress markers in rabbits treated with CCL_4_ induced hepatotoxicity as indicated in the figure. All data were expressed as mean ± SEM. Values were statistically tested using the Student* t*-test and significant differences at* p* < 0.05 and* p* < 0.01 as indicated by (*∗*)* & (∗∗*) compared to normal control, by (#)* & (##) *compared to CCL_4_ after 5 weeks, and by* (+)* &* (++)* compared to CCL_4_ after 10 weeks, respectively.

The present results showed in Table 4 & Figure 4 exert a significant decrease (P≤ 0.01) in the enzymatic activities of CAT, SOD, GR, GST, and GPx, in the liver homogenate of CCl_4_ treated rabbits 5 and 10 weeks after experimental running as compared to those of the normal control with a percentage of change (53%, 50%, 33%, 32%, 35% & 46%, 37%, 37%, 39%, and 47%, respectively). Also the present results revealed a significant increase (P≤ 0.01) in the MDA levels in the liver homogenate of CCl_4_ treated rabbit's 5 and 10 weeks after experimental running as compared to those of the normal control with a percentage of change (96% & 120%) respectively.

Daily oral administration of 200 mg/kg bw of LSS within the rabbit's diets for 5 weeks starting from day one of the experiment significantly increased (P≤ 0.01) the enzymatic activities of CAT, SOD, GR, GST, and GPx in the liver homogenate of injured rabbits with liver cirrhosis for 5 weeks as compared to those of the corresponding CCl_4_ treated group with a percentage of change (93%, 93%, 39%, 41%, 42%) and all parameters mentioned were significantly increased (P≤ 0.01) with 70%, 58%, 33%, 25%, 37%, respectively, in rabbits administrated 400 mg/kg bw of LSS and injured with liver cirrhosis for 5 weeks as compared to those of the corresponding CCl_4_ treated group. Similarly, Daily oral administration of 200 mg/kg bw of LSS within the rabbit's diets for 10 weeks starting from day one of the experiment significantly increased (P≤ 0.01) the enzymatic activities of CAT, SOD, GR, GST, and GPx in the liver homogenate of injured rabbits with liver cirrhosis with a percentage of change (70%, 58%, 52%, 58%, 74%) and all parameters mentioned were reduced significantly (P≤ 0.01) to 73%, 47%, 47%, 46%, and 65%, respectively, in rabbits administered 400 mg/kg bw of LSS and injured with liver cirrhosis for 10 weeks as compared to those of the corresponding CCl_4_ treated group. Also the results revealed that daily oral administration of 200 and 400 mg/kg bw of LSS within the rabbits' diets for 5 and 10 weeks starting from day one of the experiment significantly decreased (P≤ 0.01) the MDA levels in the liver homogenate with a percentage of change (47% & 44% and 60% & 56% for 5 and 10 weeks, respectively) compared to those of the corresponding CCl_4_ treated group (Table 4 & Figure 4).

### 3.4. DNA Results of Untreated, LSS Administered, CCl_*4*_- Treated, and LSS Protected Rabbits

**Figure 5:**
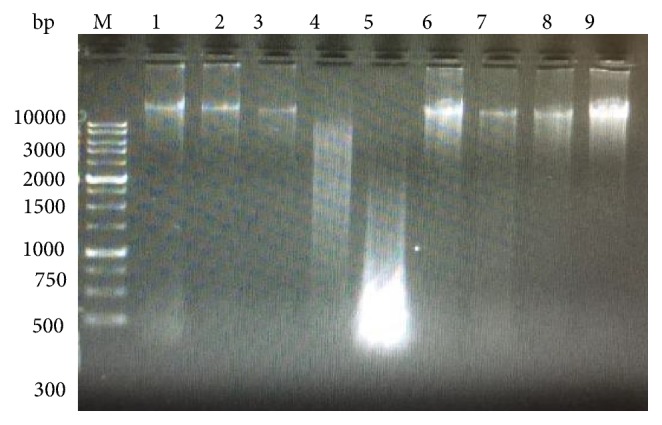
Agarose gel showing DNA damage by CCl_4_ and protective effects of various fractions of LSS. Lanes from left (M) molecular weight marker, (1) negative control, (2) LSS (200 mg/kg bw), (3) LSS (400 mg/kg bw), (4) CCl_4_ for 5 weeks, (5) CCl_4_ for 10 weeks, (6) & (7) CCl_4_ + LSS (200 mg/kg bw), (8) & (9) CCl_4_ + LSS (400 mg/ kg bw).

DNA was extracted from liver tissues of all the studied rabbits; the banding manner was observable in Figure 5. Treatment of rabbits with CCl_4_ increased significantly DNA damage of the hepatic tissues more than those of the untreated rabbits. An entirely different banding manner was detected in CCl_4_ treated rabbits which was absent from the liver tissues of the untreated rabbits. DNA extracted from 200 and 400 mg/kg bw of LSS protected rabbits' livers administered with CCl_4_ after 5 and 10 weeks of the experimental running showed a significant repair in hepatic DNA. Rabbits treated with only 200 and 400 mg/kg bw of LSS after 5 and 10 weeks of the experimental running did not display any kind of DNA fragmentation.

### 3.5. Histopathological Observations

**Figure 6:**
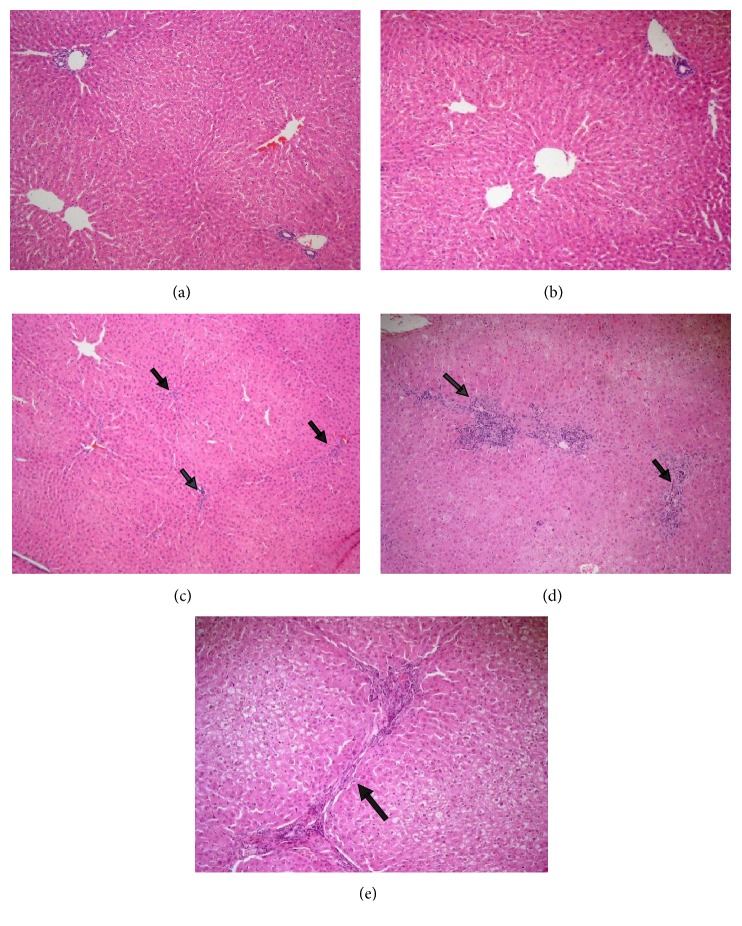
*Effect of LSS on histopathological changes of liver tissues of normal groups in rabbits*. (a) Showing normal histological picture of hepatic lobule that consists of central vein surrounded by normal hepatocytes (HEx100). (b) Showing liver sections of the rabbits treated with LSS (200 mg/kg bw.) which revealed normal histological picture of hepatic lobule that consists of central vein surrounded by normal hepatocytes (HEx100). (c) Showing liver sections of the rabbits treated with LSS (400 mg/kg bw.) which revealed normal hepatic architecture with only mild inflammation [arrows] (HEx100). (d) Showing liver sections of the rabbits treated with LSS (400 mg/kg bw.) which revealed evident chronic venous congestion and moderate inflammation [arrows] (HEx100). (e) Showing liver sections of the rabbits treated with LSS (400 mg/kg bw.) which revealed portal to portal bridging fibrosis [arrow] (HEx200).

The micrograph shown in Figure 6(a) illustrates the normal aspect of the hepatic liver for the control group (Subgroup A1) fed with normal diet without exposing to CCl_4_ hepatic intoxication. Examination of liver tissue of the control group in which rabbits received normal diet and 200 mg/kg bw of LSS in their diet on daily basis till the end of the experiment without exposing to CCl_4_ hepatic intoxication (Subgroup A2) revealed normal hepatic architecture (Figure 6(b)). Examination of the control group received normal diet and 400 mg/kg bw of LSS in their diet on daily basis till the end of the experiment without exposing to CCl_4_ hepatic intoxication (Subgroup A3) revealed only mild inflammation, except one case which showed the adverse effects of the drug, showing evident chronic venous congestion, moderate inflammation, and bridging fibrosis (Figures 6(c), 6(d), and 6(e)).

**Figure 7:**
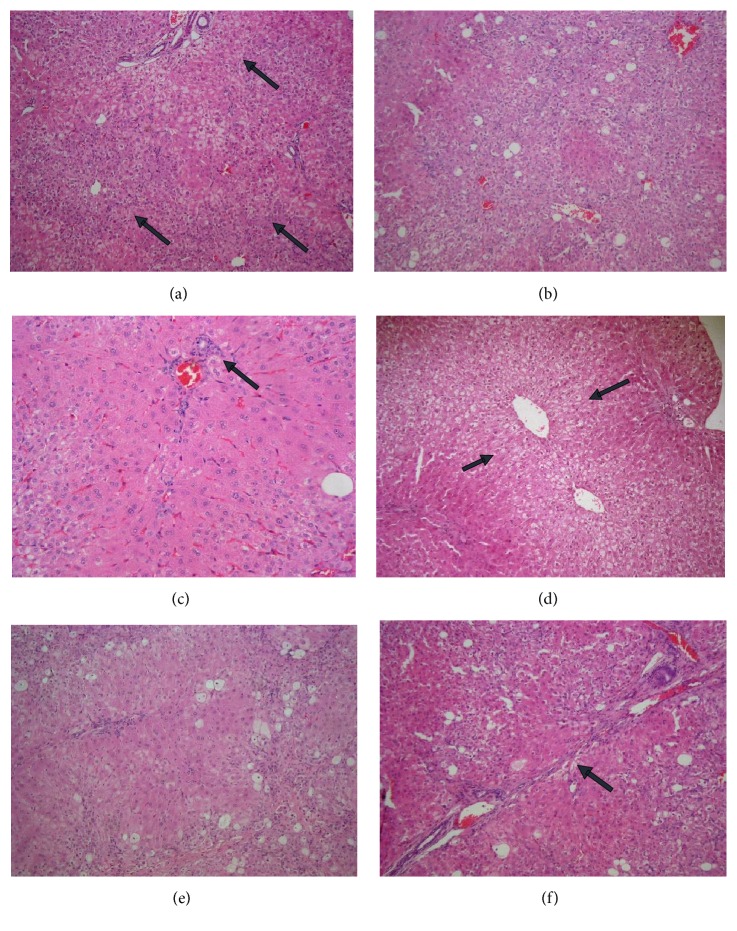
*Effect of LSS on histopathological changes of liver tissues induced by CCL4 (0.5 mL/kg i.p.)*. In rabbits sacrificed 5 weeks after experimental running as shown in (a) evident chronic venous congestion and feathery degeneration of hepatocytes [arrows] (HEx100), (b) steatosis (30%) in one case (HEx100), and (c) mild parenchymal inflammation [arrow] (HEx200). In rabbits sacrificed 10 weeks after experimental running as shown in (d) evident chronic venous congestion and feathery degeneration of hepatocytes [arrows] (HEx100), (e) steatosis (about 20%) and moderate parenchymal and portal inflammation (HEx100), and (f) bridging fibrosis [arrow] (HEx100).

According to liver tissue in rabbits exposed to CCL_4_ intoxication (Group-B1 control pollutant group), the following can be seen: hepatocyte feathery degeneration and chronic venous congestion, steatosis, parenchymal and portal inflammation and fibrosis (Figures 7(a), 7(b), and 7(c)) and (Figures 7(d), 7(e), and 7(f)), respectively.

**Figure 8:**
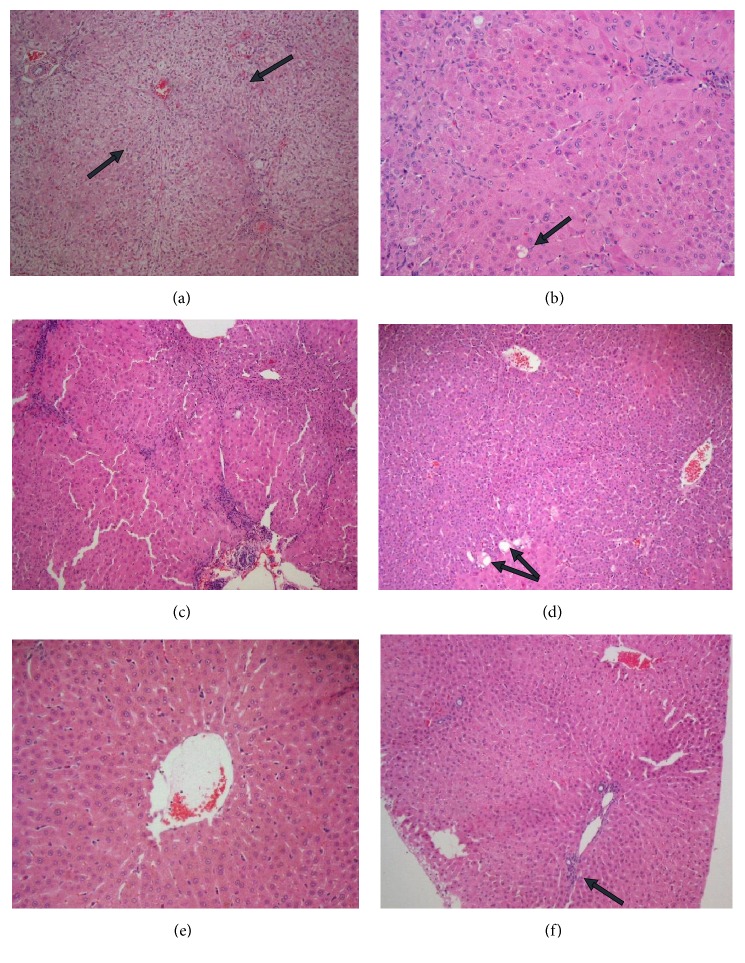
*Effect of LSLS on histopathological changes of liver tissues induced by CCL4 (0.5 mL/kg i.p.) and treated with LSS (200 mgs/kg bw)*. In rabbits sacrificed 5 weeks after experimental running as shown in (a) evident chronic venous congestion and feathery degeneration [arrow] (HEx100), (b) mild feathery degeneration and isolated vesicular steatosis [arrow] (HEx200), and (c) moderate parenchymal and portal inflammation (HEx100). In rabbits sacrificed 10 weeks after experimental running as shown in (d) regenerating hepatocytes with isolated vesicular steatosis (HEx100), (e) discrete chronic venous congestion and no steatosis (HEx400), and (f) discrete inflammatory infiltrate (HEx100).

Examination of liver tissue exposed to CCL_4_ as well as 200 mg/kg bw of LSS in their diet on daily basis (Group B2) reveals that the group which is sacrificed 5 weeks after experimental running shows evident chronic venous congestion and feathery degeneration in two cases, only mild feathery degeneration in others, no steatosis or isolated vesicular steatosis, mild parenchymal and portal inflammation (Figures 8(a), 8(b), and 8(c)). Examination of the group sacrificed 10 weeks after experimental running shows the improvement of the histoarchitectural changes of the liver towards the normal aspect, showing regenerating hepatocytes with no steatosis, discrete chronic venous congestion and discrete inflammatory infiltrate (Figures 8(d), 8(e), and 8(f)).

**Figure 9:**
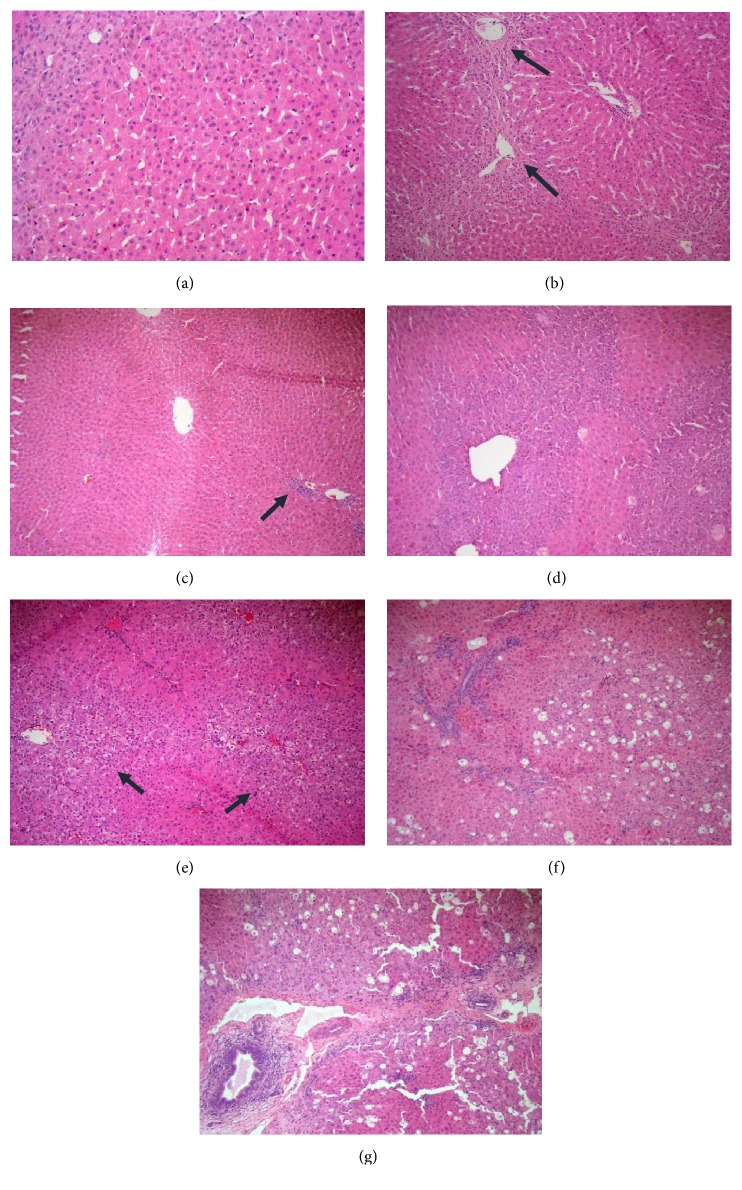
*Effect of LSS on histopathological changes of liver tissues induced by CCL4 (0.5 mL/kg i.p.) and treated with LSS (400 mgs/kg bw)*. In rabbits sacrificed 5 weeks after experimental running as shown in (a) discrete chronic venous congestion (HEx200), (b) centrilobular fibrosis [arrows], regenerating hepatocytes and isolated vesicular steatosis (HEx100), and (c) mild parenchymal and portal inflammation [arrow] (HEx100). In rabbits sacrificed 10 weeks after experimental running as shown in (d) mild chronic venous congestion and isolated vesicular steatosis (HEx100), (e) evident chronic venous congestion [arrows] (HEx100), (f) steatosis (40%) with moderate inflammation (HEx100), and (g) portal to portal bridging fibrosis in one case [arrow] (HEx100).

Examination of liver tissue of rabbits exposed to CCL_4_ as well as 400 mg/kg bw of LSS in their diet on daily basis (Group B3) reveals in the group that scarified 5 weeks after experimental running improvement of the histoarchitectural changes of the liver as there is no chronic venous congestion or only discrete chronic venous congestion and mild feathery degeneration, no steatosis or only isolated vesicular steatosis and mild parenchymal and portal inflammation (Figures 9(a), 9(b), and 9(c)). But the group that was sacrificed 10 weeks after experimental running shows adverse effects of the drug, in the form of steatosis and chronic venous congestion which is evident in two cases with mild to moderate inflammation. Bridging fibrosis is seen in one case (Figures 9(d), 9(e), 9(f), and 9(g)).

## 4. Discussion

Hepatic disease became a significant public health concern owing to growing rate of obesity in all age populations [26]. The conservative treatments of liver problems, such as acute and chronic liver hepatitis, liver cirrhosis, and fatty liver are often insufficient due to hazardous effects initiated by hepatotoxic drugs of chemical origin [27]. Medicinal plants, owing to their abundant hepatoprotective natural ingredients, have been comprehensively investigated to treat liver disorders. In the current study, hepatoprotective efficacy of LSS was investigated via hepatotoxic classical model induced by carbon tetrachloride.

In organ and tissue systems, CCl_4_ is stimulated by cytochrome P-450 dependent mixed oxidase in the hepatic endoplasmic reticulum to procedure free radical trichloromethyl (.CCl_3_ and  .Cl3COO). This causes changes in the membrane of the endoplasmic reticulum and other cellular membranes resulting in an increase in calcium ion permeability through the plasma membrane causing severe instabilities of calcium homeostasis leading to necrotic cell death [28, 29]. CCL_4_ affects many organs causing degeneration of liver fatty layer and centrilobular necrosis [29]. This phenomenon indicates that CCl_4_ is a powerful hepatotoxic agent and may be used for the characterization of the hepatic disease treatment medicines. Elevation of liver enzymatic markers (ALT, AST, ALP, and bilirubin) and increased lipid peroxidation are further evidences of hepatotoxicity. ALT and AST that exist in the hepatocytes can certainly leak into the peripheral blood as soon as the hepatocytes are injured [29].* Lepidium sativum* seed analysis by GC/MS exhibited the presence of high concentrations of mainly *α*-linolenic acid (ALA) which is* n*−3 fatty acid, an omega-3 fatty acid found in seeds exerting its bioactivity in ameliorating the hepatic intoxication and oxidative stress including liver injury induced by alcohol [30], liver steatosis [31, 32], nonalcoholic hepatic disease [33], and parenteral nutrition-associated liver disease [34]. Pari and Jalaludeen stated that orientation of the active groups in sinapic acid increases antioxidant efficiency as metal chelators and promotes its protective efficacy against liver oxidative stress in arsenic intoxicated rats [35]. The current study showed that sinapic acid as a metal chelator may exert its biological activity in improving liver function.

*Lepidium sativum* seed extracts have proved hepatoprotective effects against CCl_4_ induced liver damage. A research study on male Albino Wistar rats demonstrated significant reduction in hepatotoxicity induced by CCl_4_ administered with 200-400 mg/kg of* Lepidium sativum* seeds extract included within the diet. Serum level of AST, ALT levels, and bilirubin levels were significantly elevated in CCl_4_ treated rats compared to those of normal control. However, a significant reduction in the levels of these parameters in* Lepidium sativum* seed extracts protected rats was observed. The hepatoprotective effect could be due to the presence of alkaloid, coumarin, flavonoids, tannin, and triterpenes which enhance antioxidant activity and reduce free radical production from CCl_4_, which is the basic triggering factor for hepatotoxicity [36, 37].

In the present study, we evaluated the hepatoprotective efficacy of* L. sativum* seeds in white male New-Zealand rabbits. The present results revealed that concurrent treatment of rabbits administered with CCl_4_ for 5 and 10 weeks with* L. sativum* seeds significantly repaired their liver injurious marker enzymes as well as bilirubin, total protein, and albumin, hence approving its hepatoprotective effect. The results are in agreement with those of Afaf et al. [12] where they stated the role of LSS as efficient hepatoprotective agent. Also, the current results are consistent with those of Mohamed and his coworkers, [38] who reported that preprotection with LSEE (*Lepidium sativum* ethanolic extract) significantly prohibited the D-GalN/LPS induced rise in hepatic enzymes (AST, ALT, *γ*-GGT, ALP, total bilirubin, LDH, and total protein) thus significantly alleviated the reduction of lipid peroxidation and repaired the antioxidant enzymatic status and total protein restoring them to normal levels. D-GalN/LPS has been widely used to examine the mechanisms underlying acute liver failure [39, 40]. The hepatoprotective significance of LSEE could be due to downregulation of cytokines (TNF-*α*, IL-6) and stress gene (iNOS and HO-1) mRNA expression and upregulation of IL-10.* Lepidium sativum* ethanolic extract (LSEE) preprotection also improved the degree of structural damage and reduced inflammatory infiltration in hepatic cells. These outcomes established that LSEE alleviates hepatic impairments and structural injury through the decay of oxidative stress, inflammation, and apoptosis in the liver [38]. Mohamed Sakran and his colleagues [10] suggested that 5,6-dimethoxy-2′,3-methylenedioxy-7-C-*β*-D glucopyranosyl isoflavone extracted from* Lepidium sativum* seeds could improve the lipid profile and the liver functions in serum and reduce the free radicals generation by enhancing the antioxidant defense mechanism. New suggested isoflavonoid can be used as a probable antioxidant against paracetamol hepatotoxicity with its antioxidant characteristics and could restore the hepatic functions towards normalization [10].

In the present study, we found that improved cholesterol and triglycerides levels in* L. sativum* seeds protected rabbits as compared to CCl_4_ treated rabbits also support the hepatoprotective effect of LSS. These results can also be correlated to those of Shukla et al. [41] who reported the role of* L. sativum* seeds as hypolipidemic agent. Also the results of the present study agreed with those of Abdulrahman et al. [20] whose results showed that* L. sativum* seeds have hepatoprotective activity that could be due to its antioxidant activity, together with the presence of anti-inflammatory ingredients in* L. sativum* seeds extract.

The results of the current study revealed that the exposure to CCl_4_ for 5 and 10 weeks led to a significant decrease in the enzymatic activities of CAT, GPx, GR, GST, and SOD in the liver homogenate of CCl_4_ treated rabbits 5 and 10 weeks after experimental running compared to those of the normal control. The concurrent protection of rabbits exposed to CCl_4_ for 5 and 10 weeks with 200 & 400 mg /kg bw LSS significantly repaired their antioxidant status and reduced significantly the MDA levels, hence confirming its hepatoprotective efficacy.

Although the precise molecular mechanism of action of LSS is not completely understood, some researchers [42] have confirmed the role of LSS in inhibiting oxidative stress associated liver damage. The antioxidant activity of LSS could be due to the presence of phenolic compound and flavonoids in these seeds as have been evidenced through the preliminary screening study on LSS extract using HPLC. Our results match with those of Lee et al. [42] who reported that phenolic compounds play an essential role against oxidative stress related liver damage.

A unique expectation of the current study is to confirm that CCl_4_ induces liver DNA injury that was assessed using gel electrophoretic method. The results of the current study showed that CCL_4_ induces DNA injury, as presented by a significant progression in liver DNA disintegration of CCl_4_ treated rabbits above untreated control in the gel electrophoresis.

Lipid peroxidation of the nuclear membrane may be directly accompanied with nuclear DNA damage [43]. Nuclear DNA is concomitant with particular regions of the nuclear membrane [44]. The close proximity of nuclear DNA to the nuclear membrane can ease the interaction of genomic DNA with the peroxyl radicals and further reactive intermediates formed during membrane lipid peroxidation [43]. In the presence of transition metals, the hydroxyl radicals generated by CCL_4_ autoxidation can trigger peroxidation of the nuclear membrane lipids by metal catalyzed Haber-Weiss reaction; the lipid peroxides levels and intermediate free radicals could then be augmented by a chain reaction. Subsequently, the reactive species oxidize nuclear DNA, when the lipid peroxidation reaction occurs in the nuclear membrane, since the nuclear membrane controls the transport of messenger RNA into the cytoplasm and supports the nuclear division process, nuclear membrane peroxidation of lipids and proteins. Moreover, oxidative nuclear DNA damage could disrupt these serious cellular functions [43].

DNA findings showed that DNA disintegration is a direct result after CCl_4_ treatment in rabbits' liver tissues which was reduced by participation of antioxidants through administration of 200 mg & 400 mg/kg bw* L. sativum* seeds. Nearly complete repair of DNA damage was significant after treating rabbits with 200 mg & 400 mg/kg bw* L. sativum* seeds. The protective effect of* L. sativum* seeds on hepatic DNA injury may be due to the antioxidant activity of* L. sativum* seeds via direct scavenging of free radicals or interfering with free radicals generation.

Concerning histopathological evaluation in our study, rabbits' livers exposed to CCL_4_ show portal and parenchymal inflammatory infiltrate, feathery and ballooning degeneration, fatty change, and fibrosis. Our data are consistent with Rajeswary et al. [45] who reported dilatation of sinusoids, fatty change, disarrangement of normal hepatocytes, and centrilobular necrosis. Also A. S. Bernacchi et al. [46] reported that CCL_4_ leads to liver necrosis and fat accumulation in the rabbit.

The present study revealed that concurrent treatment of rabbits administered with CCl_4_ for 5 and 10 weeks with LSS improved their liver histopathological architecture in the form of less inflammation, regenerating hepatocytes, no steatosis or isolated vesicular steatosis, no chronic venous congestion or just discrete congestion and no fibrosis, hence approving its hepatoprotective effect. The results are in agreement with Afaf et al. [12] and Abdulrahman et al. [20] who observed the improvement of the histological structure in extract treated group as the histopathologic findings showed less inflammation, well restored hepatocytes and less area of necrosis as compared to severe necrosis and inflammation in CCl_4_ treated group of animals, which also supports the hepatoprotective effects of LLS. Their findings suggest the possibility of the presence of anti-inflammatory compounds in LSS extract which could also be a contributing factor towards the hepatoprotective action of LSS.

Althnaian [47] also reported that examination of hepatocytes showed mild to moderate degree of recovery. LSS attenuated the hepatic fat accumulation, decreased vacuolar degeneration, and improved the architecture of hepatocytes.

In our study, the reducing effect of LSS on fatty change caused by CCL_4_ intoxication is indicating the protective and curative role against fatty liver. Our results are supported by Shukla et al. [41] who reported that LSS significantly reduced steatosis. Their histopathological observations confirmed the curative efficacy of* L. sativum* liver damage. Microvesicular fatty changes, hepatocyte ballooning, and steatosis were markedly corrected by LSS. Also Wadhwa et al. [36] found that the severe fatty changes in the livers of rats caused by CCl_4_ were decreased in the treated groups.

In the present study, chronic venous congestion, feathery degeneration, and fatty changes show mild improvement in rabbits receiving 200mg/kg bw of LSS for 5 weeks and show moderate improvement in rabbits that received 200mg/kg bw of LSS for 10 weeks and rabbits that received 400mg/kg bw of LSS for 5 weeks. Our data are consistent with Afaf et al. [12] who observed that severe centrilobular hepatocellular vacuolation, hemorrhages, and congestion of the central veins noticed in CCL_4_ group showed mild improvement in groups that received 200 mg/kg bw of LSS and moderate improvement in groups that received 400 mg/kg bw of LSS.

On the other hand, rabbits that received 400mg/kg bw of LSS for 10 weeks showed evidence of liver toxicity in the form of venous congestion, hepatocyte feathery degeneration, fatty change, and fibrosis. These results are compatible with Bafeel and Ali [48] who reported that LSS had minimal effect on liver parenchyma if given in low doses. So, LSS could be used as food additive or supplement. Mineral content of calcium and phosphate and the presence of essential fatty acids in considerable level make LSS of beneficial nutritional value for many body organs including the liver. However, LSS in high dose showed some toxic effect on renal parenchyma represented by focal necrosis, hepatocyte apoptosis, and portal inflammatory cell infiltrate. Burow et al. [49] reported that these hazardous effects of LSS may be due to isothiocyanates reported to be one of LSS constituents.

## 5. Conclusions and Recommendations

The present study showed that LSS is a key factor in inhibition of the hazardous effects of CCl_4_. LSS treatment could significantly alleviate to a greater extent the degree of lipid peroxidation and restore the antioxidative enzymes and shift liver function to normal levels. In addition to antioxidants activity, LSS attenuated the hepatic fat accumulation, decreased vacuolar degeneration, ameliorated the degree of structural damage in the hepatic cells, and improved the architecture of hepatocytes. The current findings demonstrated that LSS have marked hepatoprotective activity. In future work, active ingredients of LSS extract should be studied in various animal models of severe toxicity induced by CCL_4_ and other hepatotoxic agents. The active ingredients of this plant are likely to be applied as one of the future drugs against acute liver toxicity.

## Figures and Tables

**Table 1:** Chemical pattern of LSS extract revealed by GC-MS analyses. R.T. = retention time, Area Pct= peak area, and Mwt= molecular weight.

No	RT	Area Pct	Compound name	Mwt (g/mol)	Chemical formula	M/Z
1	11.3902	0.0408	E-10-Methyl-11-tetradecen-1-ol acetate	268.24	C_17_H_32_O_2_	55.1
2	11.4485	5.9674	Hexadecanoic acid, methyl ester	270.256	C_17_H_34_O_2_	74.1
3	12.4277	80.2415	9,12,15-Octadecatrienoic acid, methyl ester	292.456	C_19_H_32_O_2_	79.2
4	13.2204	9.7366	cis-Methyl 11-eicosenoate	324.549	C_21_H_40_O_2_	292.4
5	13.2962	2.0426	Methyl 18-methylnonadecanoate	326.565	C_21_H_42_O_2_	74.1
6	14.0306	1.2517	Methyl 11-docosenoate	352.59	C_23_H_44_O_2_	320.4
7	14.1064	0.2947	Docosanoic acid, methyl ester	354.619	C_23_H_46_O_2_	74.1
8	14.8058	0.0701	Octadecanoic acid, 9,10-dihydroxy-, methyl ester, bis(trifluoroacetate)	330.5026	C_19_H_38_O_4_	74.1
9	14.8641	0.0826	Tetracosanoic acid, methyl ester	382.6633	C_25_H_50_O_2_	74.1
10	15.6218	0.2718	Benzoic acid, 3,5-dicyclohexyl-4-hydroxy-, methyl ester	264.3600	C_16_H_24_O_3_	57.1

**Table 2:** Chemical pattern of LSS extract of polyphenols and carbohydrates revealed by GC-MS analyses. R.T. = retention time, Area Pct= peak area, and Mwt= molecular weight.

No	RT	Area Pct	Compound name	Mwt	Chemical formula	M/Z
1	8.1846	2.9942	Glycerol, 3TMS derivative	308.6372	C_12_H_32_O_3_Si_3_	205
2	11.6107	6.6731	D-Pinitol, pentakis(trimethylsilyl) ether	555.0881	C_22_H_54_O_6_Si_5_	73
3	12.6062	4.2518	Palmitic Acid, TMS derivative	328.6052	C_19_H_40_O_2_Si	313
4	13.4058	30.3464	11-Octadecenoic acid, (Z)-, TMS derivative	354.6425	C_21_H_42_O_2_Si	339
5	13.4323	18.965	*alpha.-Linolenic acid*, TMS derivative	350.6107	C_21_H_38_O_2_Si	75
7	13.4906	2.6564	Stearic acid, TMS derivative	356.6584	C_21_H_44_O_2_Si	117
8	13.6123	1.0245	*Sinapinic acid*, 2TMS derivative	368.5722	C_17_H_28_O_5_Si_2_	368
9	14.2372	3.3369	11-Eicosenoic acid, (Z)-, TMS derivative	382.6956	C_23_H_46_O_2_Si	367
10	14.7773	2.9175	Sucrose, 8TMS derivative	919.7454	C_36_H_86_O_11_Si_8_	361
11	14.9521	1.2532	3,7,11,15-tetramethylhexadecan-1,3-diol, silylated	298.547	C_20_H_42_O	233
12	15.0368	3.2061	Sucrose, 8TMS derivative	919.7454	C_36_H_86_O_11_Si_8_	361
13	15.1003	1.4675	Behenic acid, TMS derivative	412.7647	C_25_H_52_O_2_Si	117
14	15.614	1.5318	D-(+)-Turanose, octakis(trimethylsilyl) ether	948.7867	C_37_H_89_NO_11_Si_8_	73
15	19.0612	1.2082	beta.-Sitosterol, TMS derivative	486.8878	C_32_H_58_OSi	129

**Table 3:** Liver profile of untreated, LSS administered, CCl_4_ treated, and LSS protected rabbits. Effect of LSS on serum marker enzymes ALT, AST, GGT, ALP, T.BILI, TP, ALB, TG, and CHOL in rabbits treated with CCL_4_ induced hepatotoxicity as indicated in the figure. All data were expressed as mean ± SEM. Values were statistically tested using the Student *t*-test and significant differences at *p* < 0.05 and *p* < 0.01 as indicated by (*∗*)& (*∗∗*)compared to normal control, by (#)* & (##) *compared to CCL_4_ after 5 weeks, and by *(+)* & *(++)* compared to CCL_4_ after 10 weeks, respectively.

Group	ALT (U/L)	AST (U/L)	G GT (U/L)	ALP (U/L)	T.BILI (mg/dl)	TP (g/dl)	ALB (g/dl)	CHOL (mg/dl)	TG (mg/dl)
Control	38.01 ± 4.68	27.25 ± 1.92	7.02 ± 1.24	22.02 ± 1.62	0.28 ± 0.03	6.50 ± 0.31	3.55 ± 0.17	16.32 ± 5.06	42.03 ± 4.69
LS (200mg/kg bw)	31.48 ± 6.78	29.41 ± 3.31	8.48 ± 1.20	57.02 ± 2.29*∗∗*	0.43 ± 0.03*∗*	6.40 ± 0.25	3.24 ± 0.13	32.17 ± 2.87*∗*	52.74 ± 2.79*∗*
LS (400mg/kg bw)	16.44 ± 1.46*∗∗*	20.76 ± 3.43*∗*	6.29 ± 2.29	53.08 ± 1.61*∗∗*	0.46 ± 0.03*∗∗*	5.86 ± 0.47	3.44 ± 0.10	31.61 ± 3.05*∗*	26.10 ± 3.04*∗*
CCl_4_ (5 wk.)	56.76 ± 5.46*∗∗*	62.30 ± 3.91*∗∗*	28.72 ± 2.76*∗∗*	114.83 ± 2.73*∗∗*	0.67 ± 0.04*∗∗*	4.14 ± 0.14*∗∗*	2.48 ± 0.25*∗∗*	89.21 ± 7.62*∗∗*	147.74 ± 7.44*∗*
% of Change	49	128	310	421	139	- 36	- 30	72	250
CCl_4_ (10 wk.)	67.00 ± 4.76*∗∗*	70.69 ± 6.45*∗∗*	39.78 ± 2.26*∗∗*	126.38 ± 3.71*∗∗*	0.60 ± 0.04*∗∗*	4.45 ± 0.1*∗∗*	2.61 ± 0.22*∗∗*	99.96 ± 6.16*∗∗*	163.79 ± 8.9*∗∗*
% of Change	76	159	468	474	114	- 31	- 26	5.12	2.9
CCl_4_ + LS (200mg/kg bw) 5 wk	30.62 ± 2.19##	24.90 ± 3.02##	19.62 ± 1.95 ##	59.60 ± 2.37##	0.33 ± 0.03##	6.49 ± 0.20##	3.39 ± 0.12##	51.35 ± 5.47##	89.78 ± 6.11##
% of Change	- 46	- 60	- 31	- 48	- 50	57	37	- 42	- 39
CCl_4_ + LS (400mg/kg bw) 5 wk	24.23 ± 2.91++	20.27 ± 3.09++	14.64 ± 1.73++	61.46 ± 4.16++	0.44 ± 0.03++	5.76 ± 0.62++	3.26 ± 0.20++	63.06 ± 5.24++	105.39 ± 5.62++
% of Change	- 57	- 67	- 49	- 46	- 34	39	31	- 29	- 28
CCl_4_ + LS (200mg/kg bw) 10 wk	38.04 ± 3.71##	23.90 ± 2.64##	15.81 ± 1.42##	52.59 ± 2.57##	0.38 ± 0.04##	6.36 ± 0.38##	3.38 ± 0.09##	52.36 ± 4.47##	98.70 ± 3.44##
% of Change	- 43	- 66	- 60	- 58	- 36	43	29	- 47	- 39
CCl_4_ + LS (400mg/kg bw)10 wk	40.18 ± 2.92++	25.16 ± 5.08++	22.92 ± 2.54++	79.54 ± 4.84++	0.48 ± 0.03 ++	6.35 ± 0.38++	3.34 ± 0.14++	65.57 ± 7.98++	105.13 ± 6.66++
% of Change	- 40	- 64	- 42	- 37	- 20	42	27	- 34	- 36

**Table 4:** Enzymatic activities of antioxidant enzymes and MDA of untreated, LSS administered, CCl_4_ treated, and LSS protected rabbits. All data were expressed as mean ± SEM. Values were statistically tested using the Student *t*-test and significant differences at *p* < 0.05 and *p* < 0.01 as indicated by (*∗*)* & (∗∗*) compared to normal control, by (#)* & (##) *compared to CCL_4_ after 5 weeks, and by *(+)* & *(++)* compared to CCL_4_ after 10 weeks, respectively.

Group	CAT (ng/g tissue)	SOD (ng/g tissue)	GR (ng/g tissue)	GST (ng/g tissue)	GPx (ng/g tissue)	MDA (nmol/ml)
Control	31.55 ± 1.99	28.20 ± 1.09	20.29 ± 0.99	15.37 ± 1.21	21.97 ± 1.70	6.47 ± 0.57
LS (200mg/kg bw)	31.27 ± 1.86	26.31 ± 1.55	19.08 ± 0.77	15.42 ± 0.67	21.29 ± 1.28	6.35 ± 0.45
LS (400mg/kg bw)	28.43 ± 2.04	26.76 ± 1.88	19.72 ± 1.28	15.55 ± 1.43	20.31 ± 0.74	6.35 ± 0.60
CCl_4_ (5 wk.)	14.75 ± 2.58*∗∗*	14.06 ± 1.39*∗∗*	13.58 ± 0.61*∗∗*	10.41 ± 1.08*∗∗*	14.09 ± 0.99*∗∗*	12.73 ± 1.06*∗∗*
% of Change	-53	- 50	- 33	- 32	- 35	96
CCl_4_ (10 wk.)	16.82 ± 1.57*∗∗*	17.67 ± 2.02*∗∗*	12.77 ± 1.36*∗∗*	9.40 ± 0.73*∗∗*	11.52 ± 1.82*∗∗*	14.27 ± 1.07*∗∗*
% of Change	- 46	- 37	- 37	- 39	- 47	120
CCl_4_ + LS (200mg/kg bw) 5 wk	28.46 ± 2.01##	27.20 ± 0.94##	18.92 ± 0.93##	14.72 ± 1.18##	20.09 ± 1.12##	6.67 ± 0.46##
% of Change	93	93	39	41	42	- 47
CCl_4_ + LS (400mg/kg bw) 5 wk	25.19 ± 1.77++	22.31 ± 1.40++	18.13 ± 0.94++	13.04 ± 1.31++	19.41 ± 1.92++	7.13 ± 0.72++
% of Change	70	58	33	25	37	- 44
CCl_4_ + LS (200mg/kg bw) 10 wk	28.72 ± 2.01##	27.96 ± 2.22##	19.49 ± 0.96##	14.92 ± 1.58##	20.06 ± 1.79##	5.69 ± 0.46##
% of Change	70	58	52	58	74	- 60
CCl_4_ + LS (400mg/kg bw) 10 wk	29.19 ± 1.70++	26.10 ± 1.43++	18.87 ± 1.73++	13.78 ± 0.86++	19.06 ± 1.41++	6.19 ± 0.52++
% of Change	73	47	47	46	65	- 56

## Data Availability

Data used to support the findings of this study are available from the corresponding author upon request.
